# The unfolding of iRFP713 in a crowded milieu

**DOI:** 10.7717/peerj.6707

**Published:** 2019-04-08

**Authors:** Olesya V. Stepanenko, Olga V. Stepanenko, Irina M. Kuznetsova, Konstantin K. Turoverov

**Affiliations:** 1 Laboratory of Structural Dynamics, Stability and Folding of Proteins, Institute of Cytology, Russian Academy of Science, St. Petersburg, Russian Federation; 2 Peter the Great St. Petersburg Polytechnic University, St. Petersburg, Russian Federation

**Keywords:** Protein unfolding, iRFP713, Protein aggregation, Excluded volume effect, Crowded milieu, Solvent properties

## Abstract

The exploring of biological processes *in vitro* under conditions of macromolecular crowding is a way to achieve an understanding of how these processes occur *in vivo*. In this work, we study the unfolding of the fluorescent probe iRFP713 in crowded environment *in vitro*. Previously, we showed that the unfolding of the dimeric iRFP713 is accompanied by the formation of a compact monomer and an intermediate state of the protein. In the intermediate state, the macromolecules of iRFP713 have hydrophobic clusters exposed to the surface of the protein and are prone to aggregation. Concentrated solutions of polyethylene glycol (PEG-8000), Dextran-40 and Dextran-70 with a molecular mass of 8000, 40000 and 70000 Da, respectively, were used to model the conditions for macromolecular crowding. A limited available space provided by all the crowding agents used favors to the enhanced aggregation of iRFP713 in the intermediate state at the concentration of guanidine hydrochloride (GdnHCl), at which the charge of protein surface is neutralized by the guanidine cations. This is in line with the theory of the excluded volume. In concentrated solutions of the crowding agents (240–300 mg/ml), the stabilization of the structure of iRFP713 in the intermediate state is observed. PEG-8000 also enhances the stability of iRFP713 in the monomeric compact state, whereas in concentrated solutions of Dextran-40 and Dextran-70 the resistance of the protein in the monomeric state against GdnHCl-induced unfolding decreases. The obtained data argues for the excluded volume effect being not the only factor that contributes the behavior of biological molecules in a crowded milieu. Crowding agents do not affect the structure of the native dimer of iRFP713, which excludes the direct interactions between the target protein and the crowding agents. PEGs of different molecular mass and Dextran-40/Dextran-70 are known to influence the solvent properties of water. The solvent dipolarity/polarizability and basicity/acidity in aqueous solutions of these crowding agents vary in different ways. The change of the solvent properties in aqueous solutions of crowding agents might impact the functioning of a target protein.

## Introduction

All biologically significant processes, including folding, binding of small molecules, protein-protein interactions, protein aggregation, the formation of amyloid fibrils, etc., take place in a cell in the environment crowded with biological macromolecules (Chebotareva, Filippov & Kurganov, 2015; Uversky et al., 2002; Zimmerman & Minton, 1993; Hatters, Minton & Howlett, 2002; Kuznetsova, Turoverov & Uversky, 2014; Kuznetsova et al., 2015; Minton, 2005) and low-molecular-weight organic substances such as metabolites and osmolytes (Sharp, 2015; Bai et al., 2017; Al-Ayoubi et al., 2017). It is generally accepted that the conditions of molecular crowding, under which a part of the cellular space is occupied by biological molecules and becomes unavailable to other molecules (Zimmerman & Minton, 1993; Minton, 2001), affect biological processes due to the excluded volume effect (Cheung, Klimov & Thirumalai, 2005; Minton, 1993; Tokuriki et al., 2004). This conception allows explaining not all experimental data (Mittal, Chowhan & Singh, 2015; Phillip & Schreiber, 2013). For example, the destabilization of a number of proteins is observed in the presence of inert synthetic polymers at high concentration widely used to mimic conditions of macromolecular crowding *in vitro* (Hatters, Minton & Howlett, 2002; Kuznetsova, Turoverov & Uversky, 2014; Kuznetsova et al., 2015; Minton, 2001), such as polyethylene glycol with a molecular mass of 8000 Da (PEG-8000) and Dextran-70 (MW = 70,000 Da) (Stepanenko et al., 2016a). Testing of the crowding conditions inside living cells using a FRET sensor designed as PEG modified with donor–acceptor pair of ATTO dyes also revealed that the excluded volume effect is not the dominant factor influencing the behavior of macromolecules, although its effect is enhanced under osmotic stress (Gnutt et al., 2015). This study pointed out at the heterogeneity of the crowding environment inside a living cell, which may be important for the functioning of biological macromolecules (Gnutt et al., 2015). It has been suggested that chemical interactions, termed as “soft” interactions, between biological molecules and the target protein or nucleic acid may either enhance or weaken the stabilizing effect of the excluded volume (Sarkar, Li & Pielak, 2013; Knowles et al., 2015; Daher et al., 2018). However, recent studies have revealed that understanding the effect of crowding on biological objects also requires taking into account changes in the solvent properties of aqueous solutions under these conditions. It was shown that the change in the solvent properties of water, such as dipolarity/polarizability and the ability of a solvent to donate/accept the hydrogen bond, in solutions of various osmolytes and polymeric crowding agents directly correlate with the influence of this substances on the stability of the target protein (Zaslavsky & Uversky, 2018; Ferreira et al., 2016; Ferreira, Uversky & Zaslavsky, 2017). Indeed, biological macromolecules with different physicochemical properties, for example, different proteins and nucleic acids, can respond differently to changes in the solvent properties of water (Kuznetsova, Turoverov & Uversky, 2014; Mittal, Chowhan & Singh, 2015; Zaslavsky & Uversky, 2018).

In present study, we investigated the effect of molecular crowding on the unfolding of near-infrared fluorescent protein iRFP713, engineered from bacterial phytochrome *Rp* BphP2 (Filonov et al., 2011). Near-infrared fluorescent proteins are widely used genetically-encoded optical probes for the visualization of biological processes in the cell and *in vivo* (Filonov et al., 2011; Filonov & Verkhusha, 2013; Chen et al., 2015; Krumholz et al., 2014). These two-domain proteins are composed of PAS domain (Per-ARNT-Sim repeats), bearing the conservative cysteine residue for the covalent binding of the biliverdin (BV) chromophore, a natural ligand of bacterial phytochromes (Bhoo et al., 2001), and GAF domain (cGMP phosphodiesterase/adenylate cyclase/FhlA transcriptional activator), containing the pocket for the chromophore incorporation (Wagner et al., 2007; Wagner et al., 2008). Previously iRFP713 was used as a model object for studying the protein unfolding *in vitro* induced by different chemical denaturants (Stepanenko et al., 2018). Additionally to BV chromophore, iRFP713 contains three other fluorophores —tryptophan residues that are differently distributed throughout the protein macromolecule: W109, W281, and W311 are located in the PAS domain, on the periphery of the GAF domain, and in the dimer interface, respectively. This allowed us to show the formation of a compact monomeric state at early stage of iRFP713 unfolding and the accumulation of an intermediate state of iRFP713 with a further increase in the denaturant concentration. The macromolecule in this state features the presence of hydrophobic clusters exposed on the protein surface. In this work, we revealed that this specific intermediate state of proteins can be detected by using various denaturants to realize conditions favorable for aggregation of proteins in this intermediate state. The sizes of iRFP713 in different structural states were estimated using gel permeation chromatography: the Stokes radius of the native dimer and the compact monomer of iRFP713 is equal to 37.5 ± 1.5 and 28.6 ± 0.4 Å, respectively (Stepanenko et al., 2018). The Stokes radius of iRFP713 in unfolded state is equal to 55 ± 2 Å (Uversky, 2002). In this regard, we chose as crowding agents a number of polymers with hydrodynamic dimensions comparable to those of iRFP713 in different structural states: polyethylene glycol 8,000 (PEG-8000) with an effective hydrodynamic radius of 24.5 ± 1.9 Å (Devanand & Selser, 1991), Dextran-40 and Dextran-70 with hydrodynamic radius of 44.5–50 and 58–64 Å, respectively (Granath, 1958; Venturoli & Rippe, 2005). Our results indicate that the behavior of biological molecules under conditions of molecular crowding is not always determined solely by the effect of the excluded volume.

## Materials & Methods

### Plasmids, mutagenesis, protein expression and purification

iRFP713 and its variants in the holo- and apoforms were expressed and purified as described previously (Stepanenko et al., 2014) (Supplemental Methods 1). SDS/PAGE in a 12% polyacrylamide gels was used to confirm that the purity of the target proteins was at least 95% (Laemmli, 1970). The concentrated protein was stored in 20 mM Tris/HCl buffer, 150 mM NaCl, pH 8.0. The measurements were carried out at a low protein concentration (absorbance was kept less than 0.1, which corresponds to a protein concentration of 0.14 mg/ml) in 20 mM Tris/HCl buffer, pH 8.0, 1 mM tris(2-carboxyethyl)phosphine (TCEP).

GdnHCl, GTC, TCEP, and crowding agents PEG-8000, Dextran-40 and Dextran-70 were purchased from Sigma (St. Louis, MO, USA). The concentration of GdnHCl and GTC in stock solutions was calculated by the refraction coefficient measured by the Abbe refractometer (LOMO, St. Petersburg, Russia).

### Spectrophotometric Experiments

The measurements of absorption spectra were conducted using a U-3900H spectrophotometer (Hitachi, Tokyo, Japan) with microcells 101.016-QS 5 × 5 mm (Hellma, Jena, Germany). All the measurements were carried out at room temperature.

### Fluorescence Spectroscopy

The fluorescence experiments were performed using a Cary Eclipse spectrofluorometer (Agilent Technologies, Mulgrave, Australia) with 10 × 10 × 4 mm cells (Starna, Atascadero, CA, USA, USA). The temperature at measurements was maintained at 23° C using a thermostat. Selective excitation of the tryptophan residues of a protein was performed at the long-wave absorption spectrum edge (*λ*_ex_ = 295 nm) where the absorption of the tyrosine residues is negligible. The parameter *A*, which characterizes the position and form of the fluorescence spectra, was calculated using the equation:

(1) \begin{eqnarray*}A=k{I}_{320}/{I}_{365}\end{eqnarray*}

where *I*_320_ and *I*_365_ are the fluorescence intensities at the emission wavelengths of 320 and 365 nm, respectively, and *k* is the coefficient taking into account the instrument sensitivity (Turoverov & Kuznetsova, 2003). The specific fluorescence of the BV chromophore of iRFP713 in the holoform was recorded at 713 nm (*λ*_ex_ = 690 nm). The correction of the recorded fluorescence intensity for the primary inner filter effect was made according to the approach proposed in (Kuznetsova et al., 2012; Fonin et al., 2014). The anisotropy of tryptophan fluorescence was determined as follows:

(2) \begin{eqnarray*}r= \frac{ \left( {I}_{V}^{V}-G{I}_{H}^{V} \right) }{ \left( {I}_{V}^{V}+2G{I}_{H}^{V} \right) } \end{eqnarray*}

where ${I}_{V}^{V}$ and ${I}_{H}^{V}$ are vertical and horizontal components of the fluorescence intensity excited by vertically polarized light, respectively, and $G={I}_{V}^{H}/{I}_{H}^{H}$ is the coefficient that determines the different instrument sensitivity for the vertical and horizontal components of the fluorescence light, *λ*_em_ = 365 nm (Turoverov et al., 1998).

The steady-state dependences of different fluorescent characteristics of iRFP713 and its variants on the denaturant concentration were recorded 24 h after manually mixing of the native protein (50 µL aliquot) with a buffer solution containing the desired concentration of a denaturant and a crowding agent (500 µL). Control experiments revealed no noticeable changes in the detected characteristics with an increase in the equilibration time.

### Circular dichroism measurements

Circular dichroism (CD) spectra were recorded using Jasco-810 spectropolarimeter (Jasco, Japan). The cells with path length of 1 mm and 10 mm were used for measurements of the far-UV CD spectra (in the range of 250–190 nm) and the near-UV (in the 320–250 nm range) / visible CD spectra (in the range from 810 to 320 nm), respectively. For every probe, the average of three scans of CD spectra was obtained and the signal of buffer solution was subtracted. The experimental data, recorded in the far-UV and near-UV region of the spectrum, were converted into units of the molar ellipticity per amino acid residue and into units of the molar ellipticity, respectively. The visible CD spectra are represented in units of the ellipticity.

### Determination of volume fraction of crowders

The volume fraction, occupied by a crowding-agent, was calculated on the basis of the value of the partial specific volume. For dextran-40 and dextran-70, the partial specific volume of 0.623 and 0.56 ml/g, respectively, estimated in the works (Rotta et al., 2017; Senske et al., 2014) was used. The value of the partial specific volume for PEG-8000 of 0.84 ml/g was determined by approximating the density of the solution containing the crowder of different concentrations (Wandrey, Bartkowiak & Hunkeler, 1999); the data on the density of PEG-8000 was taken from the work (Gonzalez-Tello, Camacho & Blazquez, 1994) (Supplemental Methods 2).

## Results

### The effect of crowding agents on the structure of iRFP713

**Figure 1:**
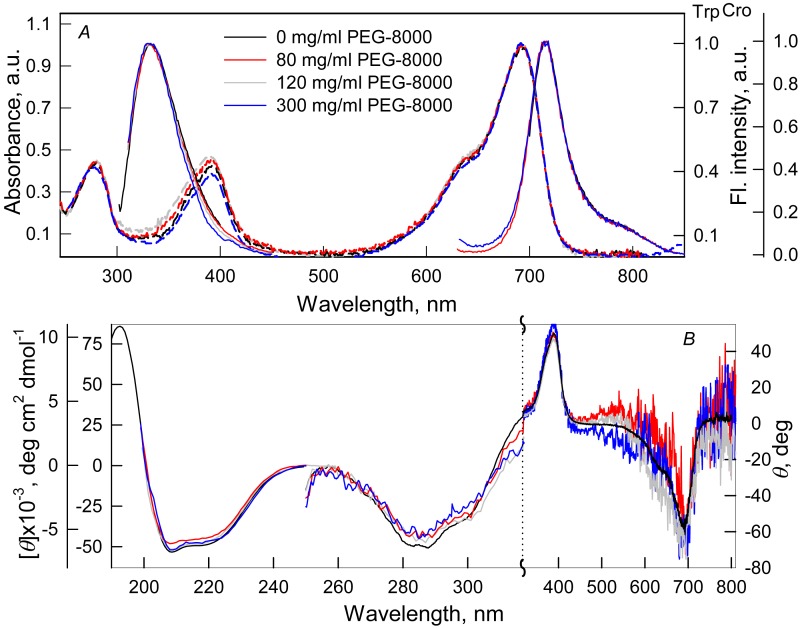
The effect of crowding agent PEG-8000 on the spectral properties of iRFP713 in the holoform. (A) Absorption spectra, tryptophan fluorescence spectra (*λ*_*ex*_ = 295 nm) and the chromophore fluorescence spectra (*λ*_*ex*_ = 690 nm). (B) CD spectra in the far-UV, near-UV and visible region of the spectrum. The color of the curves corresponds to different concentration of PEG-8000: 0 mg/ml (black line), 80 mg/ml (red line), 120 mg/ml (gray line) and 300 mg/ml (blue line). Absorption and fluorescence spectra are drawn by dashed and solid lines, respectively.

**Figure 2:**
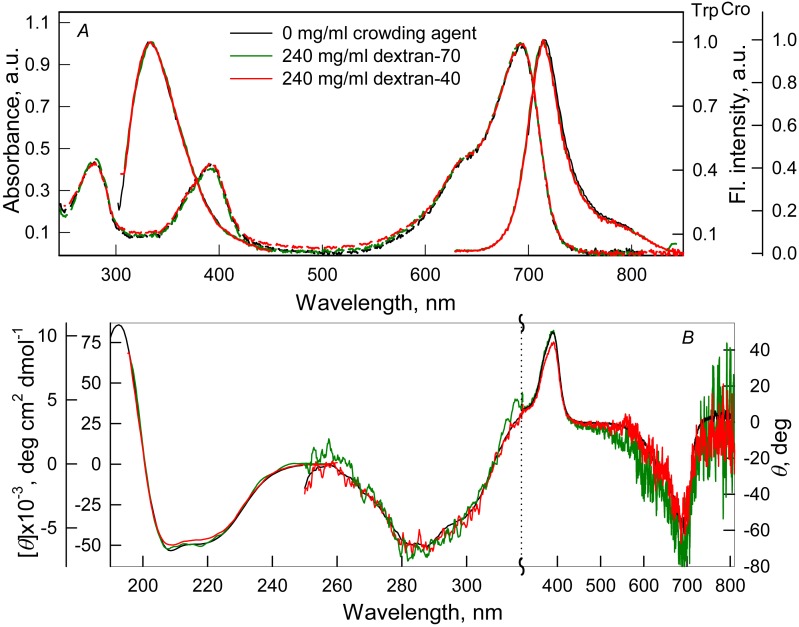
The effect of crowding agents Dextran-40 and Dextran-70 on the spectral properties of iRFP713 in the holoform. The designations on panels A–B are the same as in the caption to Fig. 1. The color of the curves symbolizes the different experimental conditions: 0 mg/ml crowding agent (black line), 240 mg/ml of Dextran-40 (red line) and 240 mg/ml of Dextran-70 (green line). Absorption and fluorescence spectra are drawn by dashed and solid lines, respectively.

We tested the effect of crowding agents Dextran-40 and Dextran-70 at a concentration of 240 mg/ml (Fig. 1) and PEG-8000 at a concentration of 80, 120 and 300 mg/ml (Fig. 2) on the structure of iRFP713 in the holoform (chromophore-bound) using absorption, fluorescence spectroscopy and circular dichroism.

Absorption spectra in the visible region, fluorescence spectra of the chromophore (Figs. 1A and 2A) and CD spectra in the visible region (Figs. 1B and 2B) recorded for iRFP713 in the holoform in solutions of Dextran-40, Dextran-70 and PEG-8000 practically coincided with the corresponding spectra of the holoprotein in the absence of a crowding agent. These data indicate that the microenvironment of the BV chromophore is preserved in the protein in the crowding conditions.

PEG-8000 at a concentration of 80–300 mg/ml and Dextran-40 and Dextran-70 at a concentration of 240 mg/ml did not affect CD spectra in the far-UV region of iRFP713 in the holoform (Figs. 1B and 2B). This testifies for the secondary structure of the protein being unchanged under these experimental conditions.

CD spectra in the near-UV region of iRFP713 in the holoform in solutions of crowding agents were almost undistinguishable from those of the protein in the absence of crowding agents (Figs. 1B and 2B). The shape of the intrinsic UV fluorescence spectra of iRFP713 in the holoform in the presence of Dextran-40, Dextran-70, PEG-8000 at a concentration of 80 and 120 mg/ml and in the absence of crowding agents coincided perfectly (Figs. 1A and 2A). The fluorescence spectrum of tryptophan residues of iRFP713 in the holoform in solutions of PEG-8000 at a concentration of 300 mg/ml deviated in the long-wavelength spectral region from the spectrum of the holoprotein in the absence of the crowder (Fig. 1A). In these experimental conditions, the value of parameter *A* of iRFP713 in the holoform was slightly higher than the value of parameter *A* of the protein in the absence of the crowding agent (Table 1). Since there observed no shift in the intrinsic UV fluorescence spectrum of iRFP713 in the holoform in the solution of PEG-8000 at high concentration relative to the position of the spectrum of the protein in the absence of the crowding agent, an increase in the parameter *A* of the protein under these conditions is hardly associated with any changes in the microenvironment of tryptophan residues. On the contrary, the small difference in the long-wavelength region of the tryptophan fluorescence spectra of iRFP713 in the holoform, recorded in the presence and in the absence of the crowding agent, is likely attributed to a high background contribution of PEG-8000 to the protein’s intrinsic fluorescence in this spectral region at increased crowder concentration. Thus, the tertiary structure of iRFP713 is preserved in crowded environment. The fact that the spatial structure of iRFP713 inherent to the native protein is unaltered under crowding conditions implies that the crowding agents used in this study do not interact with the protein. We previously demonstrated the lack of direct interaction between a number of proteins and several crowding agents, including PEG of different molecular mass, Dextran-70 and Ficoll-70 (Stepanenko et al., 2016b; Stepanenko et al., 2016c).

**Table 1:** Spectral properties of iRFP713 in the apo- and holoform in the presence of crowding agents.

	**Intrinsic fluorescence**			**Chromophore absorbance and fluorescence**			
**Conditions**	**Emission maximum (nm)**	**Parameter*A***(*λ*_**ex**_ = 295 nm)	**Fl. anisotropy,*r***(*λ*_**ex**_ = 295 nm, *λ*_**em**_ = 365 nm)	**Absorbance maximum (nm)**	**Extinction coefficient(M**^−1^**cm**^−1^**)**	**Emission maximum (nm)**	**Quantumyield (%)**
holoform of the protein							
0 mg/ml of a crowder	332	1.56 ± 0.02	0.125 ± 0.005	692	98,000	713	6.3
80 mg/ml of PEG 8000	332	1.57 ± 0.02	0.125 ± 0.005	692	96,000	713	6.3
120 mg/ml of PEG 8000	332	1.62 ± 0.02	0.13 ± 0.01	692	96,000	713	6.3
300 mg/ml of PEG 8000	333	1.80 ± 0.03	0.14 ± 0.01	692	97,000	713	6.2
240 mg/ml of Dextran-40	333	1.55 ± 0.02	0.13 ± 0.01	692	96,000	713	6.5
240 mg/ml of Dextran-70	332	1.58 ± 0.02	0.13 ± 0.01	692	97,000	713	6.1
apoform of the protein							
0 mg/ml of a crowder	332	1.50 ± 0.02	0.12 ± 0.01				
300 mg/ml of PEG 8000	334	1.70 ± 0.03	0.125 ± 0.005				
240 mg/ml of Dextran-40	333	1.51 ± 0.02	0.12 ± 0.01				
240 mg/ml of Dextran-70	334	1.53 ± 0.02	0.12 ± 0.01				

**Figure 3:**
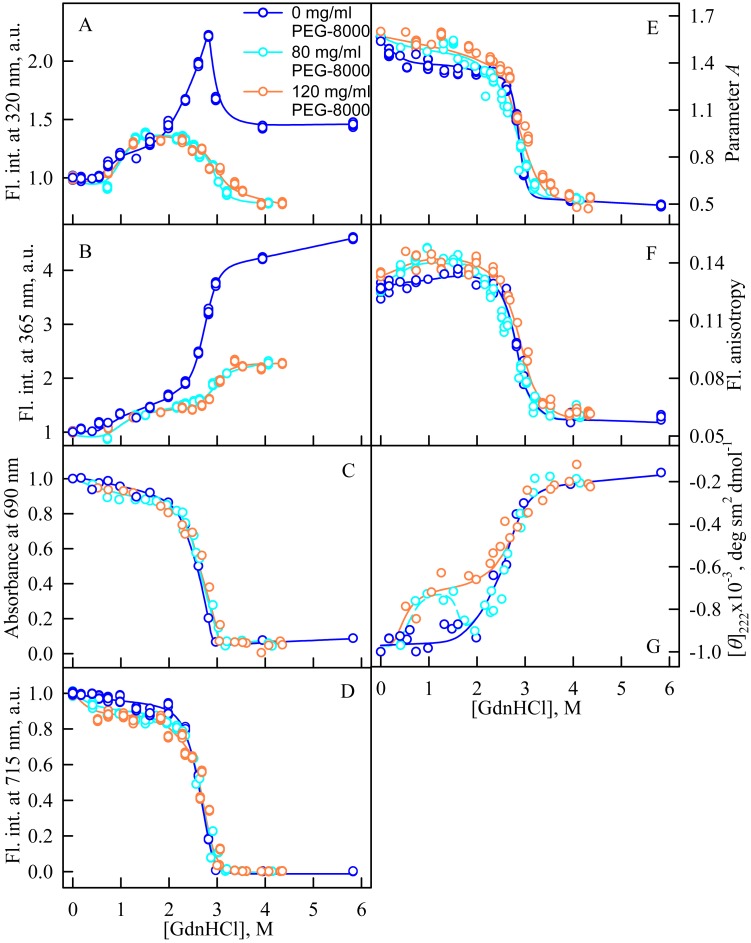
Unfolding of iRFP713 in the holoform induced by guanidine hydrochloride (GdnHCl) in the presence of PEG-8000. (A and B) Changes in the tryptophan fluorescence intensity at registration wavelengths of 320 and 365 nm. The fluorescence was excited at wavelength of 295 nm. The values of fluorescence intensity *I*_320_ and *I*_365_ were normalized to unity at zero denaturant concentration. (C) Changes in optical density of the solution; *λ* = 690 nm. (D) Changes in the chromophore fluorescence intensity at an excitation wavelength of 690 nm, corrected for the primary inner filter effect taking into account changes in the absorbance of the solution at the excitation wavelength (see Materials and Methods). (E) Changes in the parameter *A* = *I*_320_∕*I*_365_ at an excitation wavelength of 295 nm. (F) Changes in fluorescence anisotropy at excitation and emission wavelengths of 295 and 365 nm. (G) Changes in the ellipticity at 222 nm. The color of the curves corresponds to different concentration of PEG-8000: 0 mg/ml (blue circles), 80 mg/ml (cyan circles), 120 mg/ml (gray line) and 300 mg/ml (orange circles). The measurements were performed after 24 h incubation of the native protein in the presence of GdnHCl.

### Unfolding of iRFP713 in the presence of PEG-8000

**Figure 4:**
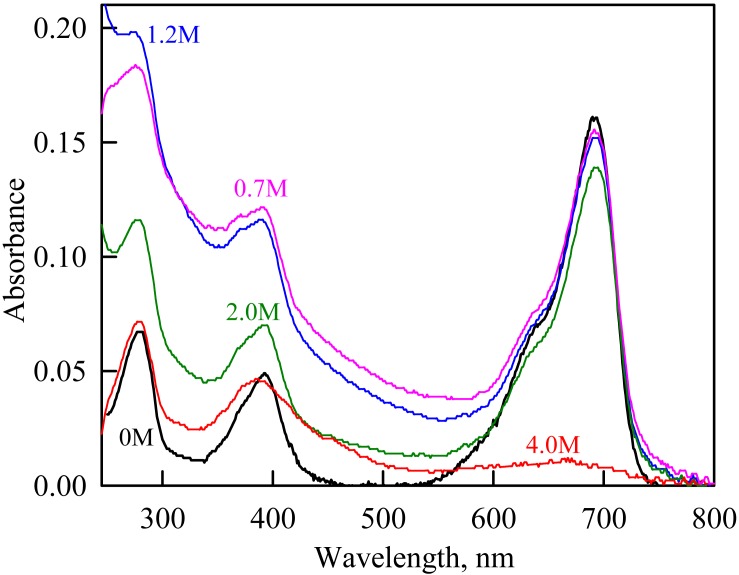
The change in the recorded absorption spectra of iRFP713 in the holoform at GdnHCl-induced unfolding in the presence of PEG-8000 at a concentration of 80 mg/ml. Numerals at the curves are the final concentration of the denaturant in the protein solutions.

We studied the unfolding of iRFP713 in its holo- and apoform (BV-free) induced by GdnHCl in the presence of 80 and 120 mg/ml of PEG-8000 (Fig. 3) and induced by GTC in the presence of 300 mg/ml of PEG-8000 (Fig. 4).

In the presence of a moderate concentration of PEG-8000 (80 and 120 mg/ml), an increase in the intensity of tryptophan fluorescence of iRFP713 in the holoform was observed in the range of GdnHCl concentration from 0.7 to 1.2 M (Figs. 3A, 3B). Under these experimental conditions, the value of the parameter *A* of iRFP713 in holoform remained almost unchanged (Fig. 3E), and the fluorescence anisotropy of tryptophan residues of the holoprotein increased slightly (Fig. 3F). In the range of GdnHCl concentration from 0.7 to 1.5 M and from 0.5 to 1.8 M in solutions of PEG-8000 at a concentration of 80 mg/ml and 120 mg/ml, respectively, a noticeable decrease in the circular dichroism signal of iRFP713 in the holoform was detected (Fig. 3G). The effect is probably caused by protein aggregation. The aggregation of iRFP713 in the holoform in crowded environment mimicked by PEG-8000 at a concentration of 80 and 120 mg/ml at denaturant concentrations less than 2 M is confirmed by the shape of the recorded absorption spectra of the protein (Fig. 4). The dependencies of the absorbance at the maximum of the far-red absorption band and of the chromophore fluorescence intensity of iRFP713 in the holoform, recorded in solutions of PEG-8000 at a moderate concentration, coincided with the dependences of the protein in the absence of the crowding agent (Figs. 3C, 3D).

When examining the dependences of the tryptophan fluorescence intensity recorded at GdnHCl-induced unfolding of iRFP713 in the holoform in the absence of a crowding agent, we found two transitions at the pre-denaturing concentrations of the denaturant: from 0.6 to 1.0 M and from 1.6 to 2.8 M (Figs. 3A, 3B). Previously, we showed that the unfolding of iRFP713 in the apo- and holoform was accompanied by the formation of a compact monomeric state of the protein and an intermediate state of the protein, bearing the hydrophobic regions on its surface (Stepanenko et al., 2018). For careful characterization of iRFP713 in these states we analyzed three mutant variants of iRFP713 containing single tryptophan residue in different domain of the protein: iRFP713-W109 (iRFP713/W281F/W311F), iRFP713-W281 (iRFP713/W109F/W311F) and iRFP713-W311 (iRFP713/W109F/W281F).

**Figure 5:**
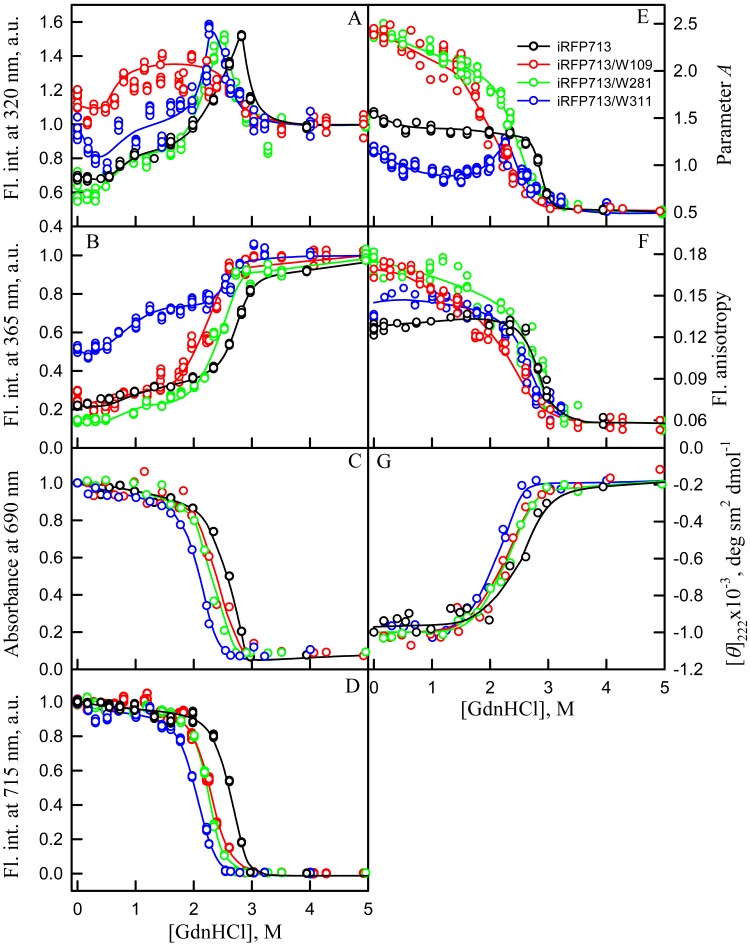
Unfolding of iRFP713 and its mutant variants in their holoforms induced by guanidine hydrochloride (GdnHCl). The designations for A–F are the same as in the caption to Fig. 3. Symbols of different color correspond to different proteins: iRFP713 (black symbols), iRFP713-W109 (red symbols), iRFP713-W281 (green symbols) and iRFP713-W311 (blue symbols). The measurements were performed after 24 h incubation of proteins in the presence of GdnHCl.

The GdnHCl-induced unfolding of single-tryptophan mutant variants of iRFP713 in the holoforms were studied (Fig. 5). The dependences of the chromophore absorbance and fluorescence intensity, and ellipticity in the far-UV region of the spectrum of all single-tryptophan mutant variants of iRFP713 in the holoforms significantly overlapped with the corresponding dependences of the iRFP713 holoprotein (Figs. 5C, 5D). This evidences that amino acid substitutions introduced into the primary sequence of iRFP713 do not have marked effect on the stability of mutant proteins.

The change of tryptophan fluorescence intensity of iRFP713-W281 and iRFP713-W311 at GdnHCl-induced unfolding was identical to the change of the characteristic of the iRFP713 holoprotein (Figs. 5A, 5B). Indeed, in the range of pre-denaturing denaturant concentrations from 0.3–0.5 to 1.0 M and from 1.5 to 2.3–2.5 M an increase in the tryptophan fluorescence intensity of iRFP713-W281 and iRFP713-W311 was observed (Figs. 5A, 5B, green and blue curves). The rise in the tryptophan fluorescence intensity of iRFP713-W311 in the range of GdnHCl concentrations corresponding to the first transition was more pronounced compared to the change in the tryptophan fluorescence intensity of iRFP713-W281 and iRFP713 (Figs. 5A, 5B, green and blue curves). The tryptophan fluorescence intensity of iRFP713-W109 also increased in the range of GdnHCl concentrations from 0.5 to 1.0 M, then remained unchanged up to denaturant concentration of 2.5 M (Figs. 5A, 5B, red curves). In the range of GdnHCl concentrations from 0.2 to 1.0 M, the parameter *A* of the iRFP713-W311 in the holoform dropped from 1.2 to 0.8 (Fig. 5E, blue curve), indicating that the tryptophan residue becomes a more accessible to the solvent, most likely as a result of the protein dimer dissociation into monomers. The value of the parameter *A* of iRFP713-W109 and iRFP713-W281 in the range of pre-denaturing GdnHCl concentration descended slightly (Fig. 5E, red and green curves), pointing out some loosening of the structure around the tryptophan residues W109 and W281. The values of absorbance and fluorescence intensity of BV chromophore of all single-tryptophan mutants in the holoforms in the range of denaturant concentration from 0.5 to 1.0 M practically did not change (Figs. 5C, 5D). Under these experimental conditions, all single-tryptophan mutants of iRFP713 retained the values of ellipticity at 222 nm close to the values of the characteristic of the proteins, registered in the absence of a denaturant (Fig. 5G). Obviously, the structure of the chromophore-binding pocket of the GAF domain and the secondary structure of the protein are preserved in iRFP713 in the monomeric state. Taken together, these data are in line with the formation of the compact monomeric state of iRFP713 at the first denaturing transition in the range of low GdnHCl concentrations. The increased fluorescence quantum yield of the tryptophan residue W311 of iRFP713 in the compact monomeric state resulted from an increase in its accessibility to the solvent at the disruption of the native dimer iRFP713 (Fig. 5E, blue curves). The increased fluorescence quantum yield of the tryptophan residues W109 and W281 of iRFP713 in the compact monomeric state may be caused by a change in the distance and mutual orientation between these tryptophan residues and quenching groups in their vicinity due to a decrease in the density of their microenvironment.

The transition in the range of denaturant concentrations from 1.5 to 2.3–2.5 M, revealed by the increase in the tryptophan fluorescence intensity of iRFP713-W281 and iRFP713-W311 in the holoforms (Figs. 5A, 5B, green and blue curves), is associated with the formation of an intermediate state of the protein. At these denaturant concentrations, the values of absorbance and fluorescence intensity of the BV chromophore of all single-tryptophan variants of iRFP713 decreased to zero (Figs. 5C, 5D). This indicates that BV chromophore is no longer embedded in the GAF domain pocket of the iRFP713 in its intermediate state, meaning the disruption of the GAF domain structure at the formation of the intermediate state of iRFP713. A decrease in the ellipticity in the far-UV region of the spectrum of all single-tryptophan variants of iRFP713 in the range of GdnHCl concentrations from 1.5 to 2.3–2.5 M testifies the loss of the secondary structure of iRFP713 in its intermediate state (Fig. 5G). The increased fluorescence quantum yield of the tryptophan residues W281 and W311 of iRFP713 at the intermediate state is explained by the absence of the conditions for the effective nonradiative energy transfer from tryptophan residues of the protein to the chromophore (Stepanenko et al., 2014; Stepanenko et al., 2017). The fact that the formation of the intermediate state was not accompanied by any increase in fluorescence intensity of iRFP713-W109 may be connected with less efficient nonradiative energy transfer from this residue to the chromophore compared to the other tryptophan residues, as W109 is the most distant tryptophan residue from the pocket of the GAF domain of iRFP713 (Stepanenko et al., 2017). An increase in the parameter *A* of iRFP713-W311 in the holoform, which was observed in the range of GdnHCl concentrations from 1.5 to 2.3–2.5 M, is obviously related with the re-shielding of this tryptophan residue in the intermediate state of iRFP713 (Fig. 5E, blue curves). The latter implies the oligomerization of iRFP713 in its intermediate state.

Thus, an increase in the intensity of tryptophan fluorescence of iRFP713 in the holoform in the range of GdnHCl concentrations from 0.6 to 1.0 M in the absence of crowding agents and from 0.7 to 1.2 M in solutions of PEG-8000 at a concentration of 80 and 120 mg/ml is associated with the formation of the compact monomeric state of the protein. PEG-8000 at a moderate concentration induced no shift of the dependences of the tryptophan fluorescence intensity of iRFP713 in the holoform in the region of this transition (Figs. 3A, 3B). This indicates that at crowded environment created by PEG-8000 at a concentration of 80 and 120 mg/ml, the structure of the native dimer of iRFP713 in the holoform is not stabilized or destabilized.

In the region of the denaturation transition, the values of all recorded characteristics of iRFP713 in the holoform in the solutions with the moderate concentration of PEG-8000 were indistinguishable from the values of the corresponding characteristics of the protein in the absence of the crowder (Figs. 3A–3G). This evidences that PEG-8000 at a concentration of 80 and 120 mg/ml does not have a noticeable stabilizing or destabilizing effect on iRFP713 in the compact monomeric or intermediate state.

As we mentioned before the apparent aggregation of the iRFP713 holoprotein was observed in the presence of PEG-8000 at a moderate concentration in the pre-denaturing range of GdnHCl concentrations (Fig. 3G). The shape of the recorded absorption spectra of iRFP713 under these experimental conditions implies that the protein aggregates most strongly at 1.2 M GdnHCl (Fig. 4). We showed previously that at the concentration of GdnH^+^ of about 1.0 M the surface charge of iRFP713 in the intermediate state is neutralized, leading to the protein aggregation (Stepanenko et al., 2018). PEG-8000 at a moderate concentration did not affect the unfolding of iRFP713 in the holoform. The GdnHCl-induced unfolding of iRFP713 in the holoform was highly cooperative (Fig. 3D). At the same time, a monotonic decrease in the fluorescence intensity of the iRFP713 chromophore was registered in the range of GdnHCl concentrations from 0 to 2.3 M (Fig. 3D) indicative of the accumulation of a small amount of the holoprotein in its intermediate state at the treatment with small denaturant concentrations. We assume that this small fraction of iRFP713 in the intermediate state is responsible for observed protein aggregation in the presence of the crowder in the range of GdnH^+^ concentrations that are optimal for the neutralization of the charge of the protein surface. Crowded milieu is known to promote protein aggregation by the excluded volume effect (Minton, 2000; Ellis & Minton, 2006).

In the presence of PEG-8000 at a high concentration (300 mg/ml) the solubility of GdnHCl decreases that makes impossible to prepare a solution of GdnHCl with a concentration above 3M. We used a stronger denaturing agent, GTC, to unfold iRFP713 in the apo- and holoform in concentrated solutions of PEG-8000.

**Figure 6:**
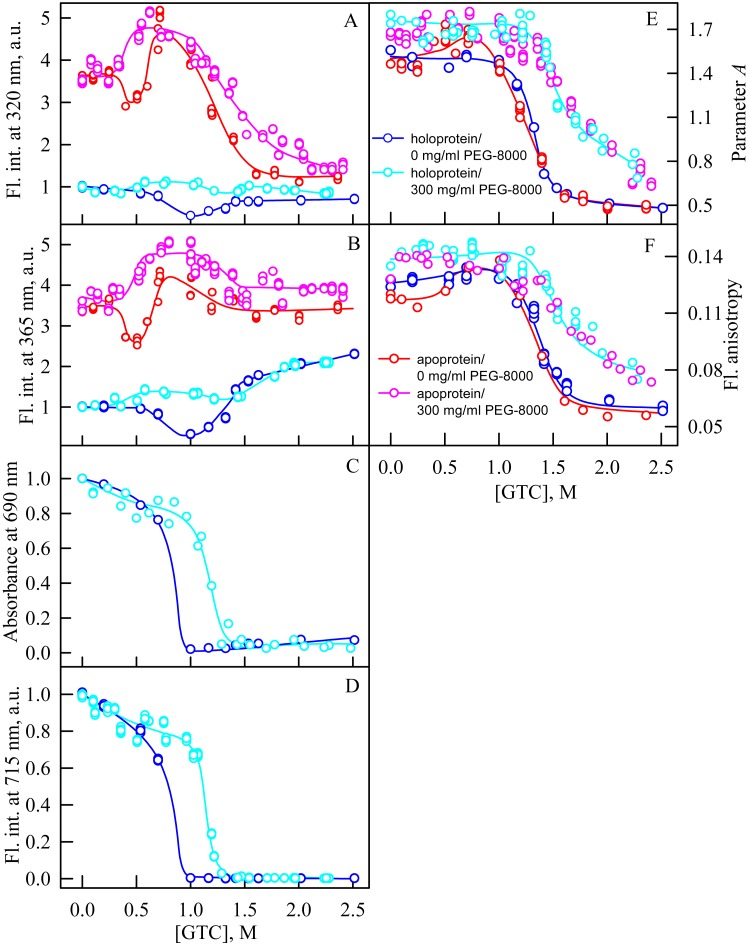
Unfolding of iRFP713 in the apo- and holoform induced by guanidine thiocyanate (GTC) in the presence of PEG-8000 at a concentration of 300 mg/ml. The designations for A–F are the same as in the caption to Fig. 3. The values of fluorescence intensity *I*_320_ and *I*_365_ of the holoprotein were normalized to unity at zero denaturant concentration. The values of fluorescence intensity *I*_320_ and *I*_365_ of the apoprotein were normalized to a value that is equal to the ratio of the fluorescence intensity at the corresponding registration wavelength of apo- and holoprotein at zero denaturant concentration. The color of the symbols indicates the concentration of PEG-8000 in solutions of apo- and holoprotein: 0 mg/ml (red and blue circles, respectively), 300 mg/ml (pink and cyan circles, respectively). The measurements were performed after 24 h incubation of apo- or holoprotein in the presence of GdnHCl.

In the presence of PEG-8000 at a high concentration, the intensity of tryptophan fluorescence of iRFP713 in the apo- and holoform increased in the range of GTC concentrations from 0.3 to 0.5 M and from 0.35 M to 0.6 M, respectively, as a result of the accumulation of the compact monomeric state of the protein (Figs. 6A, 6B). We demonstrated previously that iRFP713 in the intermediate state in the presence of GTC was capable of forming large aggregates that precipitated, which was manifested as local minima on the dependences of tryptophan fluorescence intensity of apo- and hololoprotein at a denaturant concentration of 0.4 and 1.0 M, respectively. We did not find such local minima on the dependences of the intensity of tryptophan fluorescence in the case of GTC-induced unfolding of iRFP713 in the apoform in the presence of a high concentration of PEG-8000 (Figs. 6A, 6B). The local minimum, arising from the protein aggregation, on the dependences of the intensity of tryptophan fluorescence in the case of GTC-induced unfolding of the iRFP713 holoprotein under these experimental conditions was shifted to about 1.2–1.4 M GTC and was much less pronounced than the local minimum found on the dependences of tryptophan fluorescence intensity of the holoprotein in the absence of a crowder (Figs. 6A, 6B). The aggregation of iRFP713 at GTC-induced unfolding in concentrated solutions of PEG-8000 is proved by the shape of the absorption spectra of iRFP713 in the holoform recorded in the presence of the crowding agent in the range of GTC concentrations from 0.4 to 1.8 M, with the strongest protein aggregation being observed at 1.2 M GTC. The absence of pronounced minima on the dependences of the tryptophan fluorescence intensity of iRFP713 in the apo- and holoform in the presence of PEG-8000 at a high concentration can be explained by two reasons. First, the high viscosity of concentrated solutions of PEG-8000 can slow down the sedimentation of the protein aggregates. Secondly, these experimental conditions might be less favorable for aggregation of the protein compared to its unfolding in the absence of a crowder.

In the range of GTC concentrations from 1.0 to 1.3 M in the presence of PEG-8000 at a high concentration, the absorbance and fluorescence intensity of the BV chromophore of iRFP713 in the holoform dropped to zero. The shift of the denaturation transition in concentrated solutions of PEG-8000 to higher denaturant concentrations testifies to the stabilization of the compact protein monomeric state (Fig. 5C, 5D). Stabilization of iRFP713 in the monomeric compact state in concentration solutions of PEG-8000, which hydrodynamic dimensions are comparable to the size of the iRFP713 monomer, is consistent with the theory of excluded volume (Kuznetsova, Turoverov & Uversky, 2014; Tokuriki et al., 2004). As a result of the stabilization of the compact monomeric state of iRFP713 in concentrated solutions of PEG-8000, the formation of the aggregation-prone intermediate state of the protein occurred at higher concentrations of the denaturant. It further confirmed by the shift of the local minimum on dependences of the tryptophan fluorescence intensity of the iRFP713 holoprotein, recorded in the presence of the crowder at a concentration of 300 mg/ml, to higher GTC concentration (1.2–1.4 M) compared to that of the protein, unfolded in the absence of a crowder (Fig. 5A, 5B).

In the presence of PEG-8000 at a high concentration, the unfolding of iRFP713 in the holo- and apoform, accompanied by a decrease in the parameter *A* and the fluorescence anisotropy, occurred at higher GTC concentrations than in the absence of a crowder (Figs. 5E, 5F). This indicates the stabilization of the structure of iRFP713 in its intermediate state under these conditions.

### Unfolding of iRFP713 in the presence of Dextran-40 and Dextran-70

**Figure 7:**
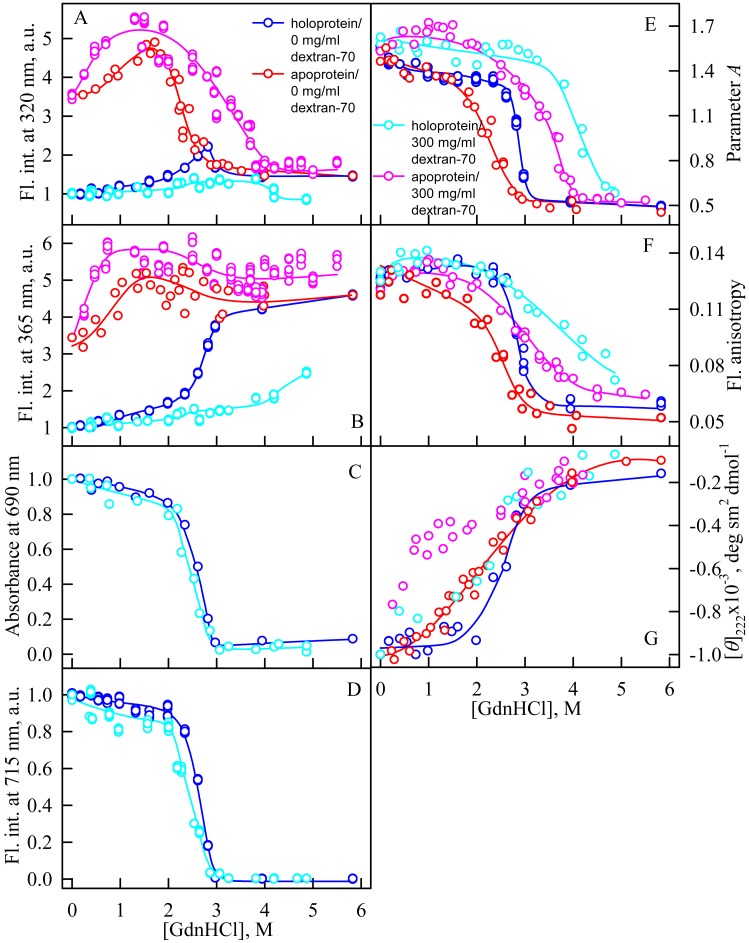
Unfolding of iRFP713 in the apo- and holoform induced by guanidine hydrochloride (GdnHCl) in the presence of Dextran-70. The designations for A–G are the same as in the caption to Fig. 3. The color of the symbols denotes the concentration of dextran-70 in solutions of apo- and holoprotein: 0 mg/ml (red and blue circles, respectively), 240 mg/ml (pink and cyan circles, respectively). The measurements were performed after 24 h incubation of apo- or holoprotein in the presence of GdnHCl.

**Figure 8:**
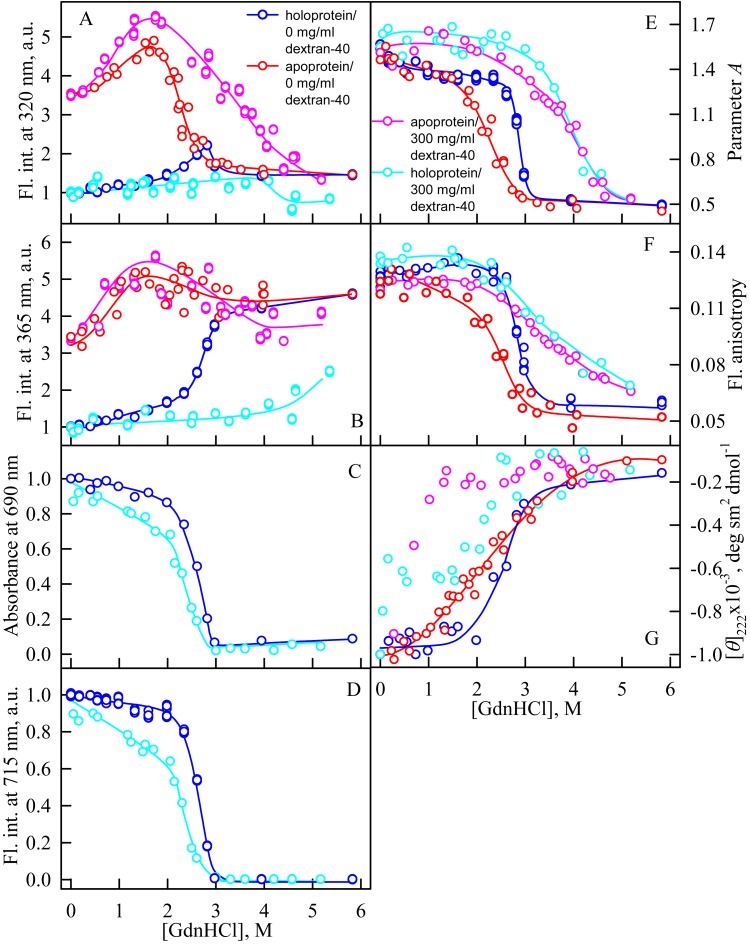
Unfolding of iRFP713 in the apo- and holoform induced by guanidine hydrochloride (GdnHCl) in the presence of Dextran-40. The designations for A–G are the same as in the caption to Fig. 3. The color of the symbols denotes the concentration of dextran-40 in solutions of apo- and holoprotein: 0 mg/ml (red and blue circles, respectively), 240 mg/ml (pink and cyan circles, respectively). The measurements were performed after 24 h incubation of apo- or holoprotein in the presence of GdnHCl.

In the presence of Dextran-70 at a concentration of 240 mg/ml, the cooperativity of the denaturing transition followed by the change in absorbance at the maximum of the far-red absorption band and the chromophore fluorescence intensity of iRFP713 in the holoform decreased (Figs. 7C, 7D). In the presence of Dextran-40 at a concentration of 240 mg/ml, this effect was even more pronounced (Figs. 8C, 8D). These data show that Dextran-70 and, particularly, Dextran-40 destabilize the structure of iRFP713 in the compact monomeric state.

In the presence of Dextran-70, the intensity of tryptophan fluorescence of iRFP713 in the holoform increased by about 40% in the range of GdnHCl concentrations from 2.0 to 2.8 M compared to the fluorescence intensity of the protein at zero denaturant concentration (Figs. 7A, 7B). Small changes in the tryptophan fluorescence intensity of iRFP713 in the holoform, recorded at the protein unfolding in the presence of Dextran-40, make their interpretation unfeasible (Figs. 8A, 8B). In the range of denaturant concentrations above 2.0 M, the absorbance and the fluorescence intensity of the BV chromophore of iRFP713 in the holoform dropped to zero in the presence of both crowding agents (Figs. 7C, 7D and 8C, 8D). Based on the above data, the denaturing transition at the GdnHCl concentrations from 2.0 to 2.8 M can be associated with the formation of the intermediate state of iRFP713. The change in the intensity of tryptophan fluorescence of the iRFP713 holoprotein occurring in the range of GdnHCl concentrations above 3.8 M in the presence of Dextran-70 is caused by the unfolding of the protein (Figs. 7A, 7B). In this range of GdnHCl concentrations, a significant decrease in the parameter *A* and the fluorescence anisotropy of iRFP713 in the holoform was detected in the presence of both crowders (Figs. 7E, 7F and 8E, 8F). The dependences of parameter *A* and fluorescence anisotropy, recorded at unfolding of iRFP713 in the apoform, were also shifted to higher concentrations of GdnHCl in the presence of both crowders compared to that of apoprotein in the crowder’s absence (Fig. 7E, 7F and 8E, 8F). This argues for the stabilization of the intermediate state of iRFP713 in the presence of Dextran-70 and Dextran-40.

**Figure 9:**
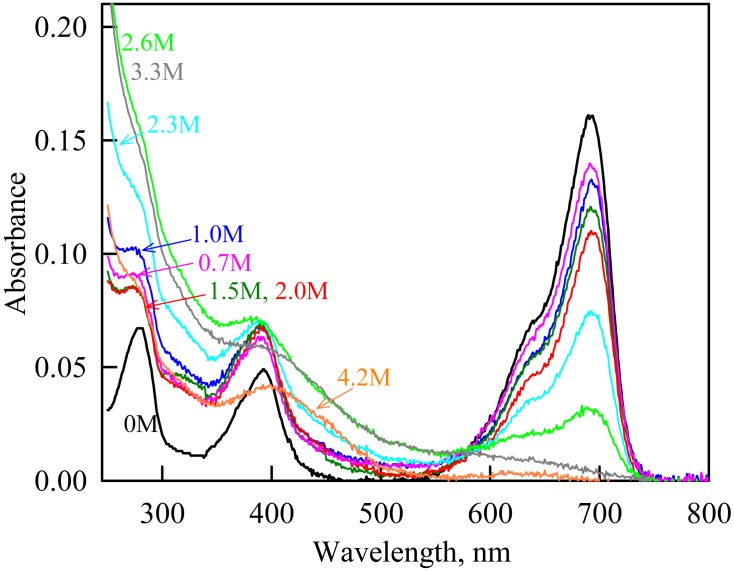
The change in the recorded absorption spectra of iRFP713 in the holoform at GdnHCl-induced unfolding in the presence of Dextran-40 at a concentration of 240 mg/ml. Numerals at the curves show the final concentration of the denaturant in the protein solutions.

In the range of pre-denaturing concentrations of GdnHCl, a noticeable decrease in molar ellipticity in the far-UV CD spectra of the iRFP713 holoform in solutions of both crowding agents was detected (Figs. 7G and 8G). Examination of the recorded absorption spectra of the iRFP713 in the holoform under these experimental conditions revealed that protein aggregated in a wide range of denaturant concentrations. Two ranges of GdnHCl concentrations were identified in which the iRFP713 in the holoform strongly aggregated in the presence of Dextran-70 and Dextran-40: about 1.0 M and about 2.6 M (Fig. 9). Probably, the aggregation of the iRFP713 holoprotein in the presence of Dextran-40 contributes to the character of the dependences of the tryptophan fluorescence intensity, recorded at these conditions (Figs. 8A, 8B). In the range of GdnHCl concentrations of about 1.0 M, the aggregation of iRFP713 in the holoform in crowded environment is facilitated by the conditions that are suitable for the neutralization of the surface charge of the protein in the intermediate state. In the range of GdnHCl concentrations of about 2.6 M, the aggregation of the iRFP713 holoprotein is related to an increase in the portion of the protein in the intermediate state. The unfolding of iRFP713 in the apoform in concentrated solutions of Dextran-70 and Dextran-40 resulted in even stronger aggregation of the apoprotein with respect to the holoprotein (Figs. 7G and 8G). Thus, the CD spectrum in the far UV region of the iRFP713 apoprotein in the presence of 1.3 M GdnHCl and of 240 mg/ml of Dextran-40 can not be detected (Fig. 8G). The enhanced aggregation of the apoprotein compared to the holoprotein in crowded environment is obviously determined by its lower stability.

## Discussion

Crowding agents PEG-8000, Dextran-40 and Dextran-70 differently affect the unfolding of iRFP713 *in vitro*. The presence of PEG-8000 at a concentration of 80–120 mg/ml, at which the volume fraction occupied by the crowding agent is 7–10%, does not influence the unfolding of iRFP713. However, the decrease of the available space in these conditions promotes the aggregation of iRFP713 in the intermediate state in the range of GdnHCl concentrations at which the GdnH^+^ ions neutralize the surface charge of the protein macromolecule. Increasing the concentration of PEG-8000 to 300 mg/ml (the volume fraction occupied by the crowding agent is 25%) leads to the stabilization of the structure of iRFP713 in monomeric compact and intermediate states. In the presence of Dextran-40 and Dextran-70 at a concentration of 240 mg/ml, destabilization of the structure of the compact monomer of the protein is observed, while the stability of iRFP713 in the intermediate state increases under these conditions. In solutions of Dextran-40 and Dextran-70, the aggregation of iRFP713 in the intermediate state is also enhanced. The volume fraction occupied by Dextran-40 and Dextran-70 at a concentration of 240 mg/ml is approximately the same and is equal to 15 and 13%, respectively. At the same time, the aggregation of iRFP713 in the intermediate state is more pronounced in solutions of Dextran-40 compared to the protein aggregation observed in solutions of Dextran-70. The theory of the excluded volume predicts that a polymer molecule of a smaller or comparable size to that of the test protein should have the greatest impact on the stability of this protein (Rivas & Minton, 2016; Minton, 1983; Hall & Minton, 2003). Stabilization of iRFP713 in the monomeric compact as well as in the intermediate states in the presence of PEG-8000 at a concentration of 300 mg/ml is consistent with the theory of the excluded volume. However, Dextran-40 and Dextran-70 at a concentration of 240 mg/ml, which hydrodynamic dimensions are larger than those of iRFP713 in the monomeric compact state, do affect the stability of the structure of the later by reducing it. All crowding agents used in this study, regardless of their size, stabilize iRFP713 in the intermediate state when applied at high concentration. Dextran-40 and Dextran-70 at high concentration have a similar stabilizing action on the structure of iRFP713 in the intermediate state, despite the fact that the hydrodynamic dimensions of Dextran-70 obviously exceed the size of the protein in the intermediate state. The obtained data indicate that the other factors in addition to the excluded volume effect contribute to the change in the stability of various structural states of iRFP713 in the presence of used crowding agents. Recent studies showed that polymers of PEG and Dextran stabilize the proteins mainly enthalpically rather than by exclusion volume effect (Senske et al., 2014). The proposed mechanism of protein stabilization in the presence of PEG and Dextran is water-mediated osmolyte-like preferential exclusion of the crowders from the protein surface. The macromolecules of PEG can additionally be involved in non-specific attractive hydrophobic interactions with a protein resulting in its enthalpic destabilization. The effect of PEG was shown to be strongly pH-dependent as it changes the charge of the protein surface and, therefore, the balance between stabilizing and destabilizing enthalpic impact of PEG (Senske et al., 2014). We have shown that the structure of the native dimer of iRFP713 is preserved in the presence of the crowding agents used in this study. Therefore, the direct interactions between the target protein in its native dimeric form and the surrounding polymer molecules can probably be ruled out in this case. The soft chemical interactions between a target protein and a synthetic polymer or protein used as a crowder are considered as affecting the behavior of biological molecules along with excluded volume effect in a number of works (Phillip & Schreiber, 2013; Zhang et al., 2012; Miklos et al., 2010). Note that the spatial structure of iRFP713 in the compact monomer is similar to those of native dimer of the protein while in the intermediate state is structurally different from both monomeric and dimeric forms of iRFP713. Thus, the possibility of interaction between crowder molecules and iRFP713 in the intermediate state cannot be excluded.

We believe that the different effect exerted by PEG-8000, Dextran-40 and Dextran-70 on the unfolding of iRFP713 can be explained by the diverse influence of these crowding agents on the solvent features of water. In a number of studies it was found that solvent properties of aqueous solutions of crowding agents change significantly compared to pure water (Ferreira et al., 2016; Ferreira, Uversky & Zaslavsky, 2017). For example, the solvent dipolarity/polarizability increases essentially in concentrated solutions of Dextran-40 and Dextran-70. In solutions of PEGs of different molecular mass (PEG-600, PEG-4500 and PEG-10000 were tested), the solvent dipolarity/polarizability remains practically unchanged, while solvent basicity, or the ability to serve as a hydrogen bond acceptor, is increased. The solvent basicity increases only slightly in aqueous solutions of Dextran-40 and even less in aqueous solutions of Dextran-70. PEGs of different molecular mass have similar impact on the solvent properties of water. It should be noted that the physicochemical properties of the iRFP713 in the compact monomeric state and the intermediate state are not identical as different amino acids are exposed to the surface of the molecule in these states. It argues for different sensitivity of iRFP713 in these structural states to the changes in the solvent properties of aqueous solutions of crowding agents.

## Conclusions

In this study, we take an advantage of the use of iRFP713 as a model object for studying the protein unfolding to investigate the effect of crowded environment on these processes. The results of the present study showed that the change of the stability of monomeric compact and intermediate states of iRFP713 in concentrated solutions of polymers used to mimic crowding conditions seems to correlate with their influence on the solvent dipolarity/polarizability and basicity/acidity. These findings confirm that the impact of the solvent properties of aqueous solutions on a target protein in a crowded milieu might be as significant as the excluded volume effect.

##  Supplemental Information

10.7717/peerj.6707/supp-1 Supplemental Information 1 Raw data presented in Figs. 1–9

10.7717/peerj.6707/supp-2 Supplemental Information 2 Detailed procedure of protein expression and purification and a description of a determination of volume fraction of crowders

## Additional Information and Declarations

### Data Availability

The following information was supplied regarding data availability: The raw data are provided in a Supplemental File.
