# Supplementary material for: The unfolding of iRFP713 in a crowded milieu
Source: PeerJ. 2019 Apr 8;7:e6707. doi: 10.7717/peerj.6707 (PMC6459179; doi:10.7717/peerj.6707)
Supplement: Supplemental Information 2 [file peerj-07-6707-s002.pdf]

SUPPLEMENTAL METHODS:

The unfolding of iRFP713 in a crowded milieu

Olesya V. Stepanenko[1], Olga V. Stepanenko[1], I.M. Kuznetsova[1], K.K. Turoverov[1, 2]

[1] Laboratory of structural dynamics, stability and folding of proteins, Institute of Cytology, Russian Academy of Sciences, St. Petersburg, Russian Federation
[2] Department of Biophysics, Peter the Great St. Petersburg Polytechnic University, St. Petersburg, Russian Federation

1. **Protein expression and purification.**

The iRFP713 genes were amplified and cloned into a pBAD/His-B vector (Invitrogen, Carlsbad, CA, USA) using BglII and EcoRI sites. LMG194 host cells (Invitrogen, Carlsbad, CA, USA) were co-transformed by pWA23h plasmid for the expression of heme oxygenase under the rhamnose promoter [1] and pBAD/His-B plasmid encoding iRFP713 and its variants with polyhistidine tags on the N-termini. Bacterial cells were grown in RM medium supplemented with ampicillin and kanamycin. The expression of heme oxygenase was initiated first by 0.02% rhamnose. After incubation of cell culture for 5 h at 37 °C the expression of the target protein was induced by 0.002% arabinose followed by the incubation of cell culture for 12 h at 37 °C and for 24 h at 18 °C. Proteins were purified with affinity chromatography on a Ni-NTA agarose column (GE Healthcare, Chicago, IL, USA). The Ni-NTA elution buffer contained 100 mM EDTA instead of imidazole. The elution buffer was exchanged to PBS buffer by dialysis. The final purification was achieved with ion-exchange chromatography on a MonoQ column (GE Healthcare, Chicago, IL, USA). The apoform of iRFP713 was expressed in LMG194 cells. The overnight LMG194 culture was grown for 2–3 h at 37 °C; then protein synthesis was induced by 0.002% arabinose. The subsequent steps of expression and purification of protein in apoform were the same as for proteins in holoform.

2. **Determination of volume fraction of crowders**

The density of the solution containing PEG-8000 at different concentration.

w^1	c (g/ml)	ρ^1 (g cm^{-3})
0.5	0.5431	1.0862
0.4	0.42684	1.0671
0.3	0.31461	1.0487
0.2	0.20634	1.0317
0.1	0.10148	1.0148
0	0	0.99707

[1] Data are taken from [2].

The partial specific volume was calculated on the basis of a linear approximation of the dependence of the density of the solution on the concentration of PEG-8000 according to the equation:

$$\rho = \rho_0 + (1 - \upsilon\rho_0)c \qquad (1),$$

where ρ_0 is the density of the solvent in the absence of crowding agent, υ is the partial specific volume and c is the concentration of a crowder.

References

1. Shcherbakova DM, Verkhusha VV (2013) Near-infrared fluorescent proteins for multicolor in vivo imaging. Nat Methods 10: 751-754. doi: 10.1038/nmeth.2521.
2. Gonzalez-Tello P, Camacho F, Blazquez G (1994) Density and Viscosity of Concentrated Aqueous Solutions of Polyethylene Glycol. J Chem Eng Data 39: 611-614. doi: 10.1021/je00015a050.
